# Self-devouring for survival: The influence of tissue-specific autophagy on seeds

**DOI:** 10.1093/plphys/kiad388

**Published:** 2023-07-05

**Authors:** Henryk Straube

**Affiliations:** Plant Physiology, American Society of Plant Biologists, Rockville, Maryland, USA; Faculty of Science, Department of Plant and Environmental Sciences, Section for Plant Biochemistry, University of Copenhagen, 1871 Frederiksberg, Copenhagen, Denmark

Seeds are a major source of calories for the global human diet. Up to 70% of the global caloric intake comes from seeds (Sreenivasulu and Wobus 2013). Storage compounds that are synthesized during seed development determine the nutritional value of seeds. Storage compounds include starch, storage lipids, and storage proteins in different proportions depending on the plant species (Sreenivasulu and Wobus 2013). Interestingly, seeds contain tissues with different parental contributions: seed coats are maternal, whereas embryos and endosperm are zygotic tissues (Bentsink and Koornneef 2008). During seed development in annual plants, the mother plant undergoes senescence, resulting in the remobilization of nutrients to the developing seeds. These remobilized nutrients are used to produce storage compounds (Distelfeld et al. 2014). Another important seed trait besides nutritional value is seed longevity, the ability of a seed to germinate after prolonged periods of dry storage. During storage, seeds deteriorate and become more susceptible to damage during germination. This eventually leads to the death of a seed (Nguyen et al. 2012). While the world's population is growing, crop productivity is stagnating (Lobell et al. 2011; Ort et al. 2015). Anthropogenic climate change is particularly challenging for seeds, highlighting the importance of understanding the effects of nutrient remobilization and seed longevity to ensure food security (Walck et al. 2011).

A process that plays an important role in plant nutrient remobilization during senescence of the mother plant is autophagy, but researchers have not distinguished between the effects of autophagy in the mother plant and in the seed itself. Autophagy refers to the degradation of cytoplasmic contents, including proteins, fatty acids, nucleic acids, and even entire organelles in plant vacuoles. The autophagy mechanism relies on highly conserved AuTophaGy-related (ATG) genes. Recent studies have shown that autophagy is involved in almost all plant developmental processes, including germination and seed development (Michaeli et al. 2016). Autophagy affects maternal tissues and embryos during seed development, particularly in N remobilization from the mother plant, because impaired autophagy pathways lead to altered C/N ratios in seeds (Guiboileau et al. 2012).

In this issue of *Plant Physiology*, Erlichman et al. (2023) differentiated the effects of autophagy in source and sink tissues. They performed reciprocal crosses of two Arabidopsis (*Arabidopsis thaliana*) *atg* mutants, *atg5-1* or *atg7-2*, with wild-type plants (Fig.). In reciprocal crosses, the parental roles are reversed to confirm previous cross results and assess the effect of parental sex on inheritance.

**Figure.**
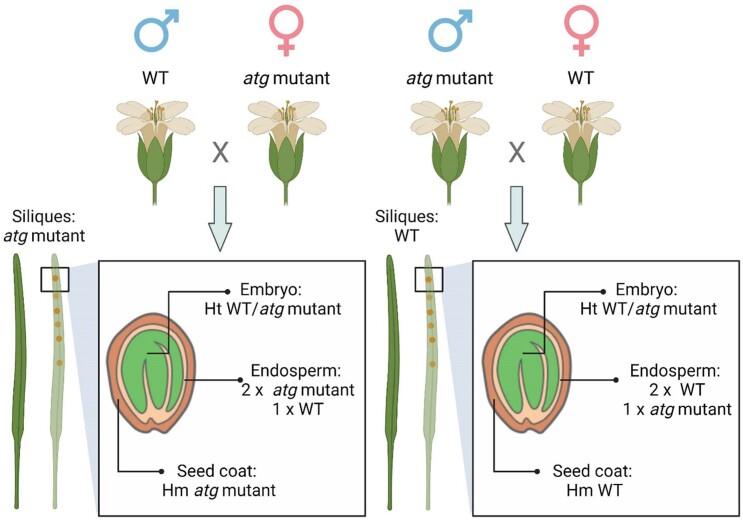
Schematic overview of the experimental system used to reveal source- and sink-specific effects of autophagy in seeds. Reciprocal crosses (WT × atg5-1, WT × atg7-2) are shown with the genetic makeup of the resulting F1 seeds and mother plants. WT, wild-type. Figure reproduced from Erlichman et al. (2023).

Reciprocal crosses between wild-type plants and the *atg* mutants (*atg*5-1 or *atg*7-2), hereafter referred to as F1 plants, possessed a partially functional autophagy mechanism because no phenotypic differences were observed under normal growth conditions. Notably, F1 seedlings from *atg* mother plants showed a reduced hypocotyl length, similar to *atg* loss-of-function mutant plants. Because previous studies have shown that *atg* mutant plants exhibit an early senescence phenotype, the authors excluded salicylic acid as the cause of the reduced hypocotyl length using crosses with plants that express the salicylic acid–degrading gene NahG.

Consistent with prior research, no differences in lipid content were detected between wild-type and mutant plants. Analyzing the protein content of F1 seeds, the researchers observed a significant reduction in total protein amount in crosses from maternal *atg7-2* plants. Although total protein content was reduced, no differences in storage protein accumulation were observed. These results suggest that autophagy in the maternal seed tissue is important for total seed protein content.

Most exciting, when Ehrlichman et al carried out germination assays, they found that F1 seeds derived from *atg* mother plants germinate faster than wild-type plants compared with F1 plants with wild-type mother plants. This is surprising because *atg* mutant seeds show delayed germination compared with wild-type seeds. Seedling establishment was unaltered regardless of genotype. Using histological techniques, the authors showed that the seed coats of *atg* mother plants had increased water permeability. Scanning electron microscopy revealed that the seeds of F1 plants derived from *atg* mother plants had misshapen seed coat cells and that the columella of these cells occupied a larger area than in the cells of wild-type plants. Because seed coat structure and permeability are important for seed longevity and aging, the authors tested the seed germination rate after artificial aging treatment. Although artificial aging resulted in overall reduced development in all lines tested, *atg* mutant lines were more strongly affected than wild-type plants.

In summary, Erlichman et al. (2023) used reciprocal crosses to dissect the effects of 2 well-studied autophagy mutants in source and sink tissues during seed development. They uncovered a specific influence of the maternal autophagy machinery on seed protein content, germination, and seed coat permeability, thereby influencing seed longevity. In future studies, it would be great to confirm these findings in plants with agronomical relevance and use plants with larger endosperms to study autophagy in this tissue.

As the human population continues to grow, future yield increases will be necessary. These increases must be based on genetic improvement. The main option is molecular breeding based on a profound understanding of seed and plant development (Sreenivasulu and Wobus 2013). Erlichman et al. (2023) contributed to this, and future studies will benefit from the approach established here using reciprocal crosses to dissect effects in seed source and sink tissues.
